# Design and Pharmacodynamics of Recombinant Fungus Defensin NZL with Improved Activity against *Staphylococcus hyicus* In Vitro and In Vivo

**DOI:** 10.3390/ijms22115435

**Published:** 2021-05-21

**Authors:** He Liu, Na Yang, Da Teng, Ruoyu Mao, Ya Hao, Xuanxuan Ma, Jianhua Wang

**Affiliations:** 1 Gene Engineering Laboratory, Feed Research Institute, Chinese Academy of Agricultural Sciences, Beijing 100081, China; liuhe2021_5@163.com (H.L.); nana_891230@126.com (N.Y.); tengda@caas.cn (D.T.); rain_mry@126.com (R.M.); haoya@caas.cn (Y.H.); 13121259099@163.com (X.M.); 2 Key Laboratory of Feed Biotechnology, Ministry of Agriculture and Rural Affairs, Beijing 100081, China

**Keywords:** fungal defensin, peptide design, *S. hyicus*, antimicrobial mechanism, efficacy in vivo

## Abstract

*Staphylococcus hyicus* is recognized as a leading pathogen of exudative epidermitis in modern swine industry. Antimicrobial peptides are attractive candidates for development as potential therapeutics to combat the serious threats of the resistance of *S. hyicus*. In this study, a series of derivatives were designed based on the NZ2114 template with the aim of obtaining peptides with more potent antimicrobial activity through changing net positive charge or hydrophobicity. Among them, a variant designated as NZL was highly expressed in *Pichia pastoris* (*P. pastoris*) with total secreted protein of 1505 mg/L in a 5-L fermenter and exhibited enhanced antimicrobial activity relative to parent peptide NZ2114. Additionally, NZL could kill over 99% of *S. hyicus* NCTC10350 in vitro within 8 h and in Hacat cells. The results of membrane permeabilization assay, morphological observations, peptide localization assay showed that NZL had potent activity against *S. hyicus*, which maybe kill *S. hyicus* through action on the cell wall. NZL also showed an effective therapy in a mouse peritonitis model caused by *S. hyicus*, superior to NZ2114 or ceftriaxone. Overall, these findings can contribute to explore a novel potential candidate against *S. hyicus* infections.

## 1. Introduction

*Staphylococcus hyicus* is one of the opportunistic and commensal pathogens which can cause acute infections to animals and humans. It was reported that exfoliative toxins-producing *S. hyicus* is primarily known as the most common causative agent of exudative epidermitis in pigs, which occurs typically as an acute skin lesions characterized by sebaceous exudation, exfoliation, and vesicle formation. This disease with prominent morbidity and mortality occurs worldwide, resulting in considerable economic losses in the swine industry [1,2,3,4]. To date, piglets infected with *S. hyicus* are frequently treated with antibiotics, but treatment failure is common due to the emergence of drug-resistant *S. hyicus* and the limited number of antimicrobial drugs available for treating exudative epidermitis. Early studies have demonstrated that *S. hyicus* frequently develops resistance to antimicrobial agents in different countries [5,6,7]. It has been found that 76.8% of isolated *S. hyicus* (*n* = 207) were resistant to penicillin and ampicillin, followed by erythromycin (56%), trimethoprim-sulfamethoxazole (28.5%), chloramphenicol (24.2%), kanamycin (19.8%) and doxycycline (1.4%) in Japan [8]. Therefore, antimicrobial peptides (AMPs) with various advantages such as broad-spectrum antimicrobial activity, non-or low resistance of bacteria and multi-target antibacterial mechanisms were different from conventional antibiotics, which deserved to be tapped for developing new therapeutic alternatives [9].

It is known that AMPs are amphiphilic cationic peptides (a few are anionic peptides, e.g., Dermicidin) with 10–100 amino acids, usually rich in arginine and lysine which are produced by various organisms like animals or plants. As the first line of defense against infection, AMPs exhibit highly effective antibacterial, antifungal, antiviral, antitumor and antiparasitic effects [10,11,12]. The antimicrobial mechanisms of AMPs derived from intracellular compatibility, high sensitivity to early warning and early resistance [13]. As an innate defensing basis of anti-infection, AMPs have attracted attention due to their unique merits such as sensitive bactericidal via multiple targets, slow resistance developing in pathogens, residue-free in tissues, regulation of gut microbiota and immunoregulation, and leading growth-promoting in animals. Thus, AMPs could cover the merits of antibiotics in disease treatment and vaccines in diseases prevention, and avoid their shortcomings, such as high resistance and high variation in pathogens, and high residual effects in animals. It is known that plectasin was the first fungus defensin isolated from *Pseudoplectania nigrella*, which displayed potent activity against *Streptococcus pneumoniae* in vitro and in mice as efficaciously as vancomycin and penicillin [14,15], whereas NZ2114 is an improved derivative (D9N, M13 L and Q14R) with more powerful activity against Gram-positive bacteria including methicillin-resistant *Staphylococcus aureus* (MRSA) and lower toxicity [15,16]. Additionally, NZ2114 exhibited intracellular activity against *S. aureus* in human THP-1 monocytes in comparison with daptomycin and vancomycin, and had great therapeutic potential in several animal models infected by *S. aureus* [17,18,19,20]. However, the development of AMPs is still hindered by some obstacles, such as undesirable toxicity, sensitivity to proteases and extreme pH, high manufacturing costs and lack of rational principles for designing effective AMPs with high cell selectivity, which limit their clinical and commercial implementation as drugs [10,12,21]. Thus, many researchers have contributed to the understanding of the structure–activity relationship of AMPs in order to overcome those limitations. Although a structure-based precise prediction of activity, mode of action, and host range may not be easy to achieve and the process involves too much randomness, certain general design principles, such as site-specific mutation, truncation, hybridization and modification, have been proposed to optimize their properties [22,23]. For instance, MP1102, a variant mutated at three sites (N9Q, L13V and R14K) of NZ2114, was designed by increasing α-helicity index and hydrophobic moment, which exerted more potent activity (MIC = 0.04 to 0.23 μM) against MRSA than the parent peptide NZ2114 (MIC = 0.11 to 0.90 μM) [24]. Besides, NZ2114 derivatives (H1–H8) displayed improved activity and longer post-antibiotic effect (PAE) against *S. aureus* through substitution of His16 and His18 with Arg and Lys residues [25]. Therefore, simplification and design of variants are the optimal ways to obtain safe and efficient novel antimicrobial agents.

In our previous study, NZ2114 was successfully expressed with large-scale production in *P. pastoris* and its activity against *S. aureus* in vitro and in vivo was evaluated [16,24], it could be designed to optimize its properties against *S. hyicus* by changing physicochemical parameters (net positive charge and hydrophobicity). In this study, a new derivative of NZ2114, named NZL, was designed and expressed in *P. pastoris* X-33. In addition, its bioavailability, antibacterial mechanism against *S. hyicus* in vitro and in a mouse peritonitis model infected with *S. hyicus* were studied for the first time.

## 2. Results

### 2.1. Characterization and Expression of Peptide

To improve the efficacy of NZ2114 and to understand the structure–activity relationship of AMPs, eleven derived peptides were designed through specific site-mutation based on the parent peptide NZ2114. Sequences 1~5 were designed by increasing hydrophobicity (0.364~0.374 vs. 0.350) while keeping charges unchanged, and sequences D6~11 were designed by decreasing one positive charge, with higher hydrophobicity (0.383~0.403) than that of the parent peptide NZ2114 (0.350). The key parameters of peptides are listed in Table 1. According to the minimal inhibitory concentration (MIC) values and inhibition zone assay, NZL (sequence 6) was picked out for further studies because of the stronger activity than the other derivatives. Compared with NZ2114, NZL with three AA substitution (N9S, L13I and R14Q) decreased a net positive charge from +3 to +2 and increased hydrophobicity from 0.350 to 0.386 and instability index from 25.49 to 20.52, which may contribute to higher antimicrobial activity and stability.

**Figure 1:**
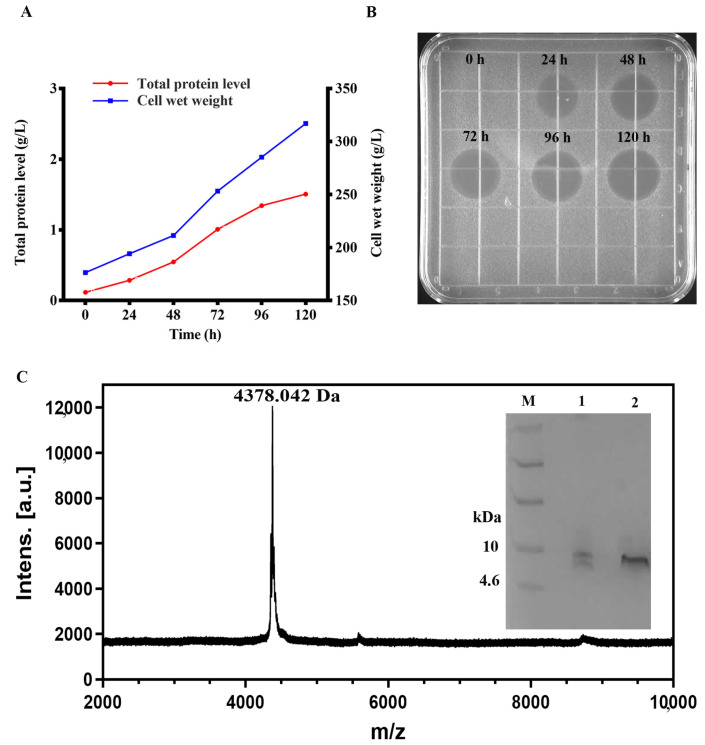
The expression of in *P. pastoris* X-33 at the fermenter level. (**A**) Time curves of the total protein levels and cell wet weights. Three samples were measured and the results were given as the mean (**B**) The inhibition zones of fermentation supernatants with different induced time against *S. aureus* ATCC 43300. (**C**) Tricine-SDS-PAGE and MALDI-TOF MS analysis of the purified NZL. M: ultra-low molecular weight protein marker. lane 1: the fermentation supernatants; lane 2: purified NZL.

The recombinant plasmids of peptides listed in Table 1 were obtained and successfully expressed in *P. pastoris* X-33. The production of peptide NZL were gradually increased with 120 h induction time in a 5-L fermenter with total secreted protein of 1505 mg/L and biomass of 317 g/L (Figure 1A) and the antimicrobial activity determined by the size of inhibition zone (Figure 1B). The target band (approximately 4 kDa) was clearly observed in gels (Figure 1C) and the molecular weight analyzed by MALDI-TOF MS was 4378.04 Da, which was close to the theoretical molecular value (4361.94 Da) (Table 1).

### 2.2. Structure Analysis

**Figure 2:**
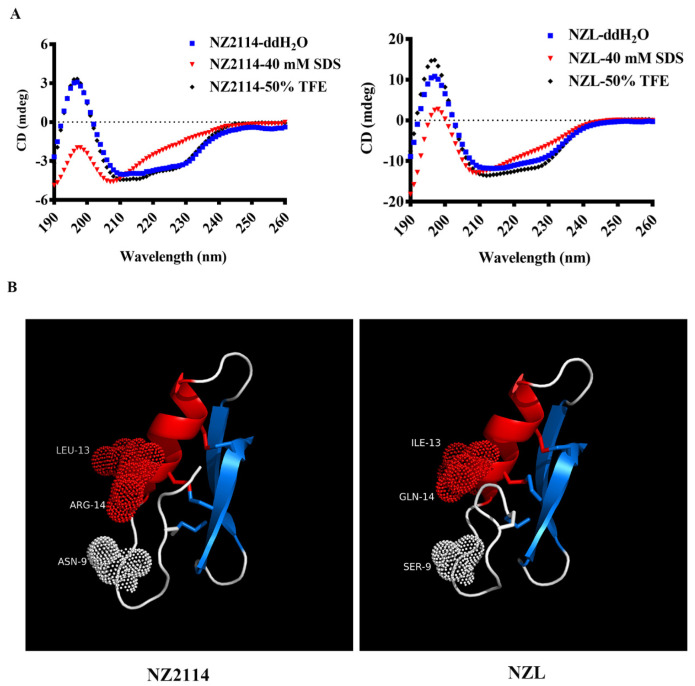
The structure analysis of NZ2114 and NZL. (**A**) CD spectra of the peptide NZ2114 and NZL in H2O, 40 mM SDS, or 50% TFE. (**B**) The three-dimensional structure molecular modeling of NZ2114 and NZL. Light dots represent mutation residues.

As shown in Figure 2A, the circular dichroism (CD) spectra showed that NZ2114 and NZL had a positive peak at 196 nm and two negative peaks at approximately 208 and 228 nm, respectively, which demonstrated that the peptides were characterized by α-helix and β-sheet or random coil in various environments [26]. However, the CD peak of NZL showed a significant increase in α-helix in 40 mM sodium dodecyl sulfate (SDS) mimicking the bacterial membrane, indicating that NZL tended to form α-helix structure and enhance membrane interactions [27]. Additionally, the three-dimensional structure modeling result predicted that similar to NZ2114, NZL possessed a typical cystine-stabilized alpha-beta structure (CSαβ) conformation, including an α-helix (residues 12–20), an antiparallel β-sheet (residues 17–31 and 36–40), and three disulfide bonds (Cys4–Cys30, Cys15–Cys37, and Cys19–Cys39) (Figure 2B).

### 2.3. Antimicrobial Activity of NZL

#### 2.3.1. MIC Determination

As shown in Table 2, NZL had potent antimicrobial activity against Gram-positive bacteria including *S. aureus* and *S. hyicus* with low MIC values (0.23~0.92 μM), superior to NZ2114 (0.91~1.81 μM) and ceftriaxone (CRO) (6.04~12.09 μM). Particularly, compared with NZ2114, the MICs of NZL against the clinical strain of *S. hyicus* ACCC 61734 increased four-fold.

#### 2.3.2. Time-Killing Curves

**Figure 3:**
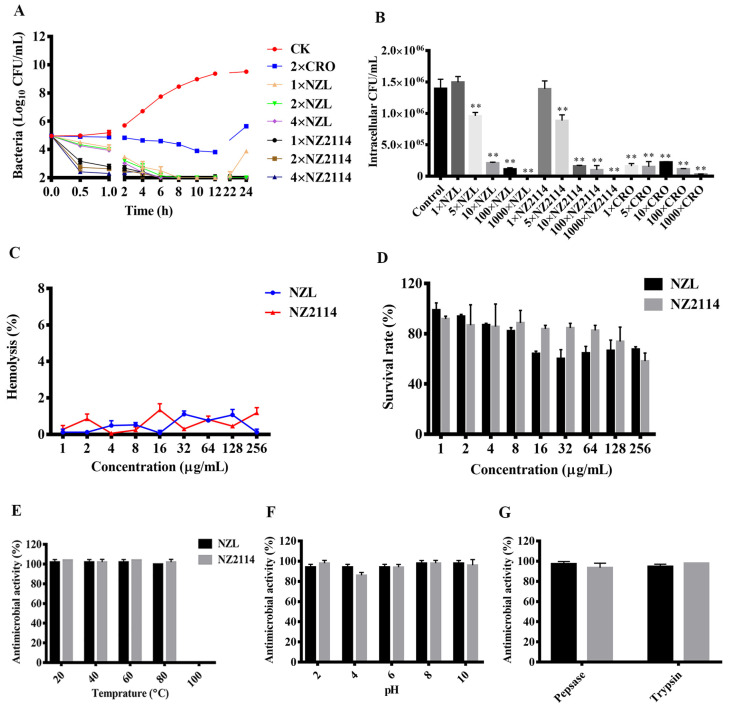
Time-kill curves, intracellular activity, toxicity and stability of NZL with NZ2114 and CRO as positive controls (**A**) Time-kill curves of NZL (1×, 2×, or 4× MIC) against *S. hyicus* NCTC 10350. CRO (2× MIC) and PBS were used as the positive and negative control, respectively. *S. hyicus* NCTC 10350 was incubated in Mueller-Hinton broth (MHB). (**B**) Intracellular activity of NZL against internalized *S. hyicus* NCTC 10350 in Hacat cells. Hacat cells were infected with *S. hyicus* NCTC 10350 and incubated with 1×, 5×, 10×, 100×, and 1000× MIC NZL, NZ2114 or CRO, respectively. (**C**) Hemolytic activity of NZL (1–256 μg/mL) against mouse erythrocytes. (**D**) Cytotoxicity of NZL (1–256 μg/mL) toward Hacat cells. Effects of temperature (**E**), pH (**F**), and proteases (**G**) on the antibacterial activity of NZL against *S. hyicus* NCTC 10350. The results were given as the mean ± SD (*n* = 3). The analyses were measured by one-way ANOVA, with Duncan’s multiple comparisons test. A probability value of < 0.05 was considered significant. (*) Indicates the significance between control and treatment groups. ** *p* < 0.01.

As shown in Figure 3A, the time-killing curves showed that after exposure to 2×, and 4× MIC of NZL or NZ2114, the bacterial counts were obviously decreased and *S. hyicus* NCTC 10350 cells were killed completely within 8 h. However, 1× MIC NZL treatment reduced bacterial counts of 7.37 Log_10_ CFU/mL, which only inhibited the growth of bacteria temporarily and bacteria regrew after 22 h. In contrast, the bacterial counts in the 2× MIC CRO-treated group showed the slowest killing rate and regrowth of bacterial cells was observed at 24 h (5.65 Log_10_ CFU/mL).

#### 2.3.3. Intracellular Antibacterial Activity

As shown in Figure 3B, NZL and NZ2114 could kill intracellular *S. hyicus* NCTC 10350 in Hacat cells in a concentration-dependent manner, CRO didn’t show a concentration-dependent manner. After exposure to 1× and 5× MIC of CRO, intracellular *S. hyicus* significantly decreased by 88.53% and 89.25%, respectively, there was no significant difference with 10× MIC of NZL (84.95%), NZ2114 (88.53%) and CRO (83.87%). The killing rate of 1000× MIC NZL, NZ2114 or CRO was up to 99.96%, 99.85% and 97.6%, respectively. Therefore, CRO showed a better intracellular killing activity than NZL and NZ2114.

### 2.4. Toxicity and Stability of NZL

As shown in Figure 3C, the maximum hemolysis of NZL against mouse erythrocytes was 1.11% at 256 μg/mL, slightly lower than that of NZ2114 (1.35%). Meanwhile, the minimum cell viability of NZL toward Hacat cells was 60.28% within 256 μg/mL, higher than that of NZ2114 (58.21%) (Figure 3D).

As shown in Figure 3E, NZL and NZ2114 maintained good thermal stability range from 20 to 80 °C, but had no activity at 100 °C. Moreover, NZL and NZ2114 retained strong stable activity in different pH values (from 2 to 10), but NZ2114 (86%) showed lower activity than NZL (94%) in the acidic environment (pH 4) (Figure 3F). Peptides are easily digested by various proteases in all bodily fluids [28]. However, NZL and NZ2114 had a desirable resistance to pepsin and trypsin (94%~98%) (Figure 3G), indicating that NZL may be delivered orally.

### 2.5. Effects of NZL and Fluorescein Isothiocyanate (FITC)-Labeled NZL on S. hyicus Membrane

**Figure 4:**
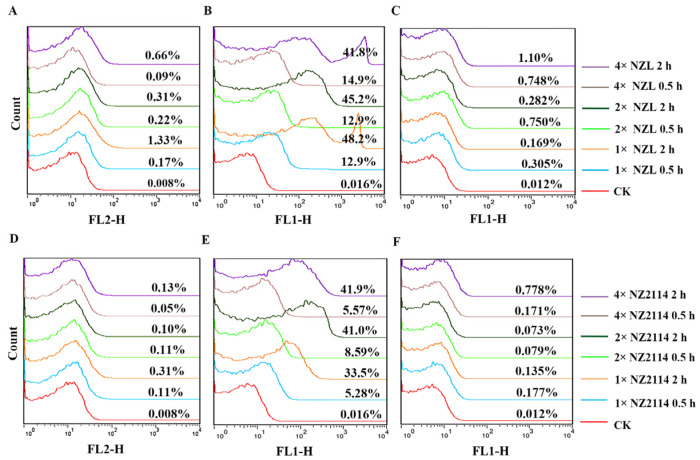
Effects of NZL and FITC-labeled NZL on *S. hyicus* membrane. FACS analysis of PI staining in *S. hyicus* cells treated with 1×, 2×, or 4× MIC NZL (**A**) or NZ2114 (**D**) for 0.5 and 2 h, respectively. FACS analysis of FITC fluorescence intensity in *S. hyicus* cells treated with 1×, 2×, or 4× MIC FITC-labeled NZL (**B**) or NZ2114 (**E**) for 0.5 and 2 h without trypan blue, respectively. FACS analysis of FITC fluorescence intensity in *S. hyicus* cells treated with 1×, 2×, or 4× MIC FITC-labeled NZL (**C**) or NZ2114 (**F**) for 0.5 and 2 h after quenching the extracellular FITC fluorescence with trypan blue, respectively.

In summary, NZL possessed potent antimicrobial activity and stability at different conditions as well as low toxicity. Therefore, the antibacterial mechanism of NZL against *S. hyicus* NCTC 10350 was further explored. Firstly, the effects of NZL and FITC-labeled NZL on *S. hyicus* membrane were evaluated. Propidium iodide (PI) can penetrate the damaged cell membrane and was adopted to evaluate the membrane integrity of *S. hyicus*. As shown in Figure 4A,D, in the untreated group, the percentage of PI-stained *S. hyicus* cells was only 0.008%. After exposure to 1×, 2×, and 4× MIC of peptides for 0.5 h or 2 h, the percentages of PI-positive cells were 0.09%~1.33% (NZL) and 0.05%~0.31% (NZ2114), indicating the cell membranes were not destroyed by peptides.

As shown in Figure 4B,E, after treatment with FITC-labeled peptides for 0.5 h or 2 h, 12.9%~48.2% of cells (NZL) showed FITC fluorescent signal, higher than those of NZ2114 (5.28%~41.9%). However, after quenching the extracellular FITC fluorescence with trypan blue, the percentages of FITC-positive cells were 0.073%~0.778% (NZL) and 0.05%~0.31% (NZ2114), approximate with the untreated group (0.012%) (Figure 4C,F), which indicated that the peptides may bind to the outside of the membrane of *S. hyicus*.

### 2.6. Morphological Observations

**Figure 5:**
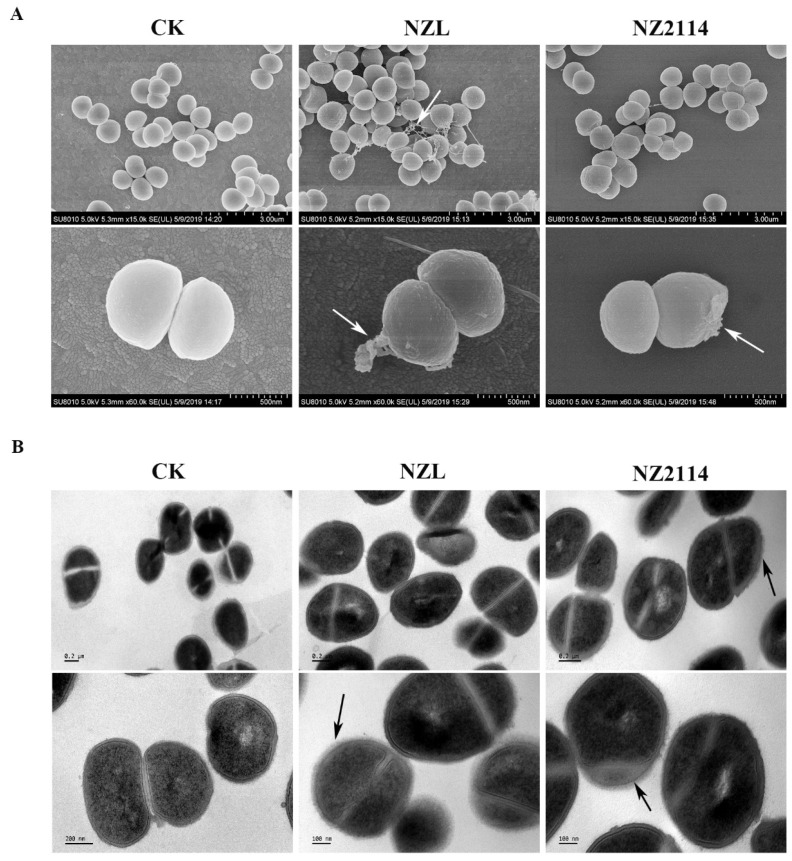
Morphological observations. (**A**) SEM images of *S. hyicus* cells treated with 4× MIC NZL or NZ2114 for 2 h. The untreated cells were used as a control group (PBS), white arrows: bubbling bulges and some filiferous adhesions. (**B**) TEM images of *S. hyicus* cells treated with 4× MIC NZL for 2 h. The untreated cells were used as a control group (PBS), black arrows: thinned and blurred cell walls.

Scanning electron microscopy (SEM) and transmission electron microscopy (TEM) were conducted to observe the microscopic morphology and intracellular ultrastructural changes of *S. hyicus* NCTC 10350 after treatment with peptides. In the control group, the *S. hyicus* cells exhibited a complete membrane morphology and a dense internal structure (Figure 5). In contrast, NZL and NZ2114 caused bubbling bulges, some filiferous adhesions on the surface of *S. hyicus*, no holes or disruption were found in *S. hyicus* cells (Figure 5A). Furthermore, as shown in Figure 5B, TEM images showed that the thinned and blurred cell walls of *S. hyicus* with light dense cellular contents were observed after treatment with NZL and NZ2114, indicating that the peptides may act on the cell wall of *S. hyicus*.

### 2.7. Super-Resolution Microscopy Image

**Figure 6:**
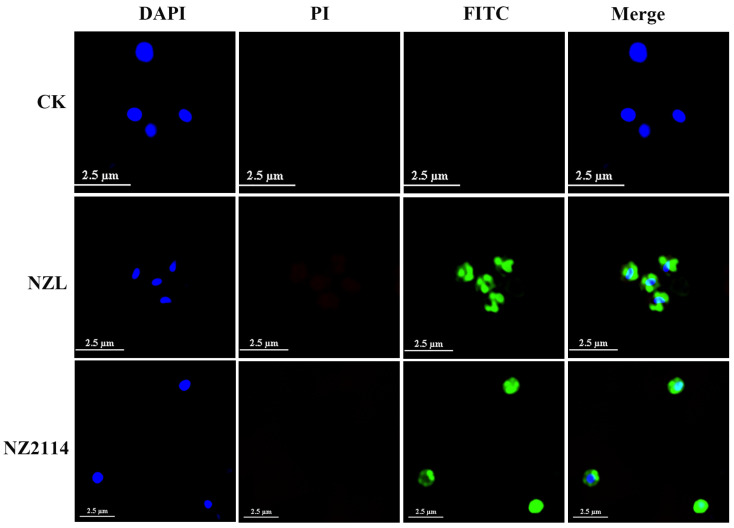
FITC-Labeled peptide interaction with the bacterial membrane. Super-resolution microscopy image analysis of *S. hyicus* treated with FITC-labeled NZL or NZ2114. Three fluorescent channels from left to right are represented DAPI, PI and FITC, respectively. The green signal is from the FITC peptide, and the blue signal is from DAPI. These graphs were from three scans per sample.

FITC-labeled peptides were adopted to preliminary detect the site of action of NZL and NZ2114. The results displayed that only the green signal (FITC) and the blue signal (4′,6-diamidino-2-phenylindole, DAPI) were observed, the green fluorescence derived from FITC-NZL or FITC-NZ2114 mainly encompassed the surface of the entire *S. hyicus* membrane, surrounding the blue fluorescence derived from nucleus (Figure 6). However, the red signal (PI) was not observed, which initially indicated that the NZL and NZ2114 positioned on the cell surface and didn’t disrupt the integrity of the *S. hyicus* membrane.

### 2.8. Efficacy of NZL in Mice

#### 2.8.1. Protection of Mice

**Figure 7:**
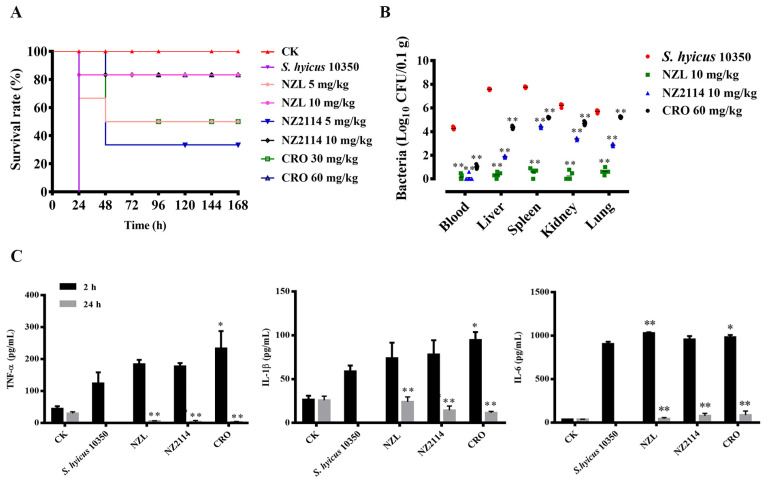
Protection efficacy of NZL in a mouse peritonitis model infected with *S. hyicus* NCTC 10350. (**A**) Survival of mice. Mice were infected intraperitoneally with *S. hyicus* NCTC 10350 (10^9^ CFU) and treated with peptides (5 mg/kg and 10 mg/kg) or CRO (30 mg/kg and 60 mg/kg) after 2 h and 8 h post infection. Survival was recorded for 7 days. (**B**) The bacterial counts of mice in the blood, livers, spleens, kidneys and lungs after treatment with peptides (10 mg/kg) or CRO (60 mg/kg). Untreated mice were used as the negative control. Data were expressed as mean ± SD (*n* = 5). (**C**) Effects of NZL on sera cytokines. Mice were challenged with *S. hyicus* NCTC 10350 (10^9^ CFU) followed by injection with peptides (10 mg/kg) or CRO (60 mg/kg). Sera were collected and the levels of TNF-α, IL-1β and IL-6 were detected by using an ELISA kit after 2 h and 24 h after treatment, respectively. The analyses were measured by one-way ANOVA, with Duncan’s multiple comparisons test. A probability value of < 0.05 was considered significant. (*) Indicates the significance between control and treatment groups. * *p* < 0.05; ** *p* < 0.01.

Therapeutic efficacy analysis was performed in a mouse peritonitis model infected with *S. hyicus* NCTC 10350. As shown in Figure 7A, all the mice were alive in the blank control, but the untreated mice died within 24 h after infection with *S. hyicus*. However, after exposure to 5 mg/kg NZL, the survival rate was 50%, superior to 5 mg/kg NZ2114 (33.3%), 10 mg/kg NZL and NZ2114 showed the same survival rates (83.3%). Although CRO presented the identical survival rates with NZL, the dose (30 mg/kg and 60 mg/kg) was significantly higher than that of NZL (5 mg/kg and 10 mg/kg).

#### 2.8.2. Inhibition of Bacterial Translocation

To identify whether intraperitoneal *S. hyicus* leads to the translocation from peritoneal cavity to deep organs, the organs were collected and homogenated at 24 h post-treatment for colony counting. As shown in Figure 7B, the bacterial counts (Log_10_ CFU/0.1 g) of the untreated mice in the blood, livers, spleens, kidneys and lungs were 4.26, 7.58, 7.75, 6.21 and 5.69, respectively. After exposure to 10 mg/kg NZL, few viable bacteria (Log_10_ CFU/0.1 g) were observed in blood (0.16), livers (0.34), spleens (0.58), kidneys (0.25) and lungs (0.62), respectively, significantly superior to those of 10 mg/kg NZ2114 (blood: 0.12, livers: 1.90, spleens: 4.41, kidneys: 3.39, and lungs: 2.87) and 60 mg/kg CRO (blood: 1.02, livers: 4.35, spleens: 5.19, kidneys: 4.69, and lungs: 5.21), which indicated that NZL showed remarkable inhibition of *S. hyicus* translocation.

#### 2.8.3. Regulation of Cytokines

The serum levels of pro-inflammatory cytokines (tumor necrosis factor-α (TNF-α), interleukin-1β (IL-1β) and interleukin-6 (IL-6)) were detected to evaluate the effects of peptides on the protective response. As shown in Figure 7C, the levels of TNF-α, IL-1β and IL-6 in the NZL-treated group from 2 to 24 h were 184.14–5.75 pg/mL, 74.00–24.02 pg/mL and 1029.28–44.92 pg/mL, respectively, and NZ2114-treated group from 2 to 24 h were 177.47–4.01 pg/mL, 78.24–14.47 pg/mL, and 958.45–78.28 pg/mL and CRO-treated group from 2 to 24 h were 233.16–3.43 pg/mL, 94.75–11.60 pg/mL, and 981.56–85.74 pg/mL, respectively. The pro-inflammatory cytokines level of NZL-, NZ2114- and CRO-treated group at 24 h were significant reduced than the untreated control groups (TNF-α: 124.10 pg/mL, IL-1β: 58.85 pg/mL, and IL-6: 905.51 pg/mL), but there was no significant difference between them. These results demonstrated that NZL, NZ2114 and CRO significantly downregulated of pro-inflammatory cytokines and protected the mice in the immune level.

#### 2.8.4. Protection of the Organ Injury

**Figure 8:**
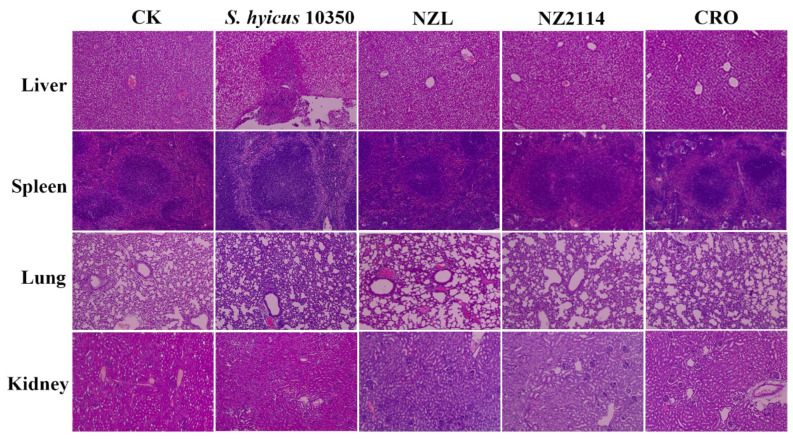
Protection efficacy of NZL on organ injury. Mice were infected intraperitoneally with *S. hyicus* NCTC 10350 (10^9^ CFU) and treated with peptides (10 mg/kg) or CRO (60 mg/kg). Livers, spleens, lungs and kidneys were harvested from mice sacrificed at 5 d after infection.

As shown in Figure 8, no obvious pathological symptoms were observed in the blank control. However, in the untreated group, the organs were damaged to a certain degree, and they were characterized by acute injury of liver tissue and inflammatory cells infiltration in the necrotic foci in liver; red pulp hemorrhage, proliferative lymphocytes in the splenic cord and enlarged splenic nodules in spleen; proliferation of alveolar septal fibroblasts and fibrin exudation from alveolar space in lung; and renal tubular atrophy and degeneration in kidney. In contrast, after treatment with NZL or NZ2114, the organs were apparently less damaged and no obvious pathological changes occurred in kidney, comparable to CRO. These results demonstrated that NZL could protect mice from a challenge with *S. hyicus*.

## 3. Discussion

It is a very common finding that *S. hyicus* isolated from exudative epidermitis-infected herds exhibits broad-spectrum resistance to antimicrobials. The presence of resistant *S. hyicus* and high morbidity rates (up to 90%) during exudative epidermitis outbreaks often make it a laborious and economically impractical task [6,7,29,30]. AMPs, as new therapeutic agents in the future, are currently under evaluation, whereas some challenges such as low antimicrobial activity and relatively high cytotoxicity limit their clinical applications [27,31]. It has been argued that the most difficult problem in the development of AMPs is that new candidates without optimization have been put into preclinical and clinical trials too quickly, thereby leading to failure, which highlights the importance of optimization in the early stages of laboratory development [32,33]. Plectasin, as the first fungal defensin, was first reported in 2005, which leads the development of AMPs [14,34]. However, the plectasin derivative NZ2114 displayed more powerful activity against *S. aureus* and was overexpressed in the yeast system, which has attracted extensive attention of scientists recently [16,20]. Therefore, new derived peptides are designed to further improve the antimicrobial activity and properties of NZ2114.

AMPs have multiple physicochemical parameters, such as length, sequence, net charge, helicity, amphipathicity, hydrophobicity and stability, whereas these parameters are intimately correlated, and alteration of one parameter can inevitably change the others, making it complicated to elucidate the impacts of a single factor on activity and toxicity [22,23]. It is generally accepted that hydrophobicity and charge are the determinant parameters of effective interaction between peptides and the microbial membranes [35]. For another, there is a critical threshold for the hydrophobicity and net positive charge, causing an increase in hemolysis once beyond a threshold [36,37]. In this study, eleven derived peptides were generated by changing net positive charge or hydrophobicity. As shown in Table 1, only sequences 6~10 possessed activity against *S. aureus* ATCC 43300 detected by inhibition zone assay, but NZL displayed the most potent activity against *S. aureus* and *S. hyicus* according to the MIC values, revealing that the correlation between bioactivity and peptide charge is complex and not linear but increases with the overall hydrophobicity within the threshold of less than 0.403 [22,27]. A previous study had demonstrated that NZ2114 derivatives, H6 and H8, with more positive charges (+5) led to the reduction of the antimicrobial activity compared with H1, H2 and H3 (+4) [25]. Speculation based on the results demonstrated that an ideal threshold of net positive charges was +2~+4. Additionally, as shown in Figure 2B, NZL maintained CSαβ structure, similar to plectasin and NZ2114 [14]. It has been confirmed that the CSαβ scaffold is a determining factor for the activity of AMPs, structural motif disruption by substituting cysteines in one pair of disulfide bonds with alanine could make it inactive [26]. NZL (Lys31) differs in only one position from NZX (Arg31), which was reported in former studies [9,38], although both Arg and Lys residues have +1 charge in neutral buffer, Arg residue has a more dispersed positive charge due to its guanidinium side chain group. The primary amine of Lys and the guanidinium group of Arg seem to interact differently with phospholipids [39]. Tryptophan fluorescence shift measurements suggested that the Arg residues strongly interacted with both zwitterionic and anionic phospholipids, whereas the Lys residues interacted weakly with zwitterionic phospholipids, but strongly with anionic phospholipids. Lys-containing peptides selective membrane interaction with negatively charged phospholipids as the main component of bacterial cell membrane displays the effective antibacterial activity, and this may explain differences in antibacterial activity between Arg-and Lys-containing peptides [39], so that best option of site modification could be designed and chosen.

The MIC value obtained is a crucial factor in the initial selection of candidate peptides [32,40]. Besides, the toxicity and stability of AMPs are the developmental obstacles for their clinical applications [27]. In this work, compared with NZ2114, NZL had lower MIC values (0.23~0.92 μM) (Table 2), hemolytic activity (1.11%) (Figure 3C) and cytotoxicity (60.28% viability) (Figure 3D), which may be related to the increase of hydrophobicity and α-helix content in 40 mM SDS mimicking the bacterial membrane (Table 1 and Figure 2A) and thereby enhancing the affinity of peptide to cell membrane [22,24]. NZL had good stability in different circumstances (Figure 3E–G), whereas NZ2114 (86%) showed lower activity than NZL (94%) in the acidic environment (pH 4), indicating that NZL is more resistant to harsh conditions than NZ2114. In addition, NZL exhibited high intracellular activity against *S. hyicus* toward Hacat cells, but it displayed significantly lower antimicrobial activity than its extracellular activity (Figure 3B). Similar to previous studies, NZ2114 and its derivative MP1102 displayed intracellular bactericidal effect against *S. aureus* toward human THP-1 monocytes and RAW 264.7 macrophages, respectively, which are weaker than their extracellular activities. It may be related to acidic pH of the phagolysosomes where intracellular pathogens can adapt to acidic environment and reproduce in host cells, thus affecting the cellular uptake and antimicrobial activities of peptides [18,41,42].

Generally, it is well known that AMPs are characterized by a net positive charge and a high ratio of hydrophobic amino acids, allowing them to selectively bind to negatively charged bacterial membranes and insert into the membranes [10,12,43]. The bactericidal mechanism of lytic membrane AMPs is widely believed to be due to the formation of pores in the bacterial cytoplasmic membrane, inducing a leakage of contents and finally cell death, e.g., cecropin and arenicin analogues [44,45,46,47]. Non-lytic membrane AMPs display specific interactions with macromolecule ingredient of bacteria, which different with these generic models of pore formation [44]. Human α-defensin 1 [48], human β-defensin 3 [49] and fungal defensin plectasin and its derivative NZ2114 [15] could block bacterial cell wall biosynthesis by specific identification with lipid II as a cellular target. Antimicrobial peptide Pep-1-K kills *S. aureus* by the formation of small channels allowing ions or protons to pass through but not disrupt the membrane [50]. In this study, the results of membrane permeabilization assays (Figure 4), EM observations (Figure 5) and super-resolution microscopy images (Figure 6) were consistent, revealing that NZL maybe exserted the bactericidal effect through action on the cell wall, similar to the non-lytic membrane mechanism of NZ2114 and plectasin [15].

Importantly, NZL showed higher activity against *S. hyicus* in mice compared to NZ2114 or CRO. One of the key advantages of AMPs over pharmaceutical antibiotics is that AMPs have the ability to modulate immune responses [43]. Likewise, in this work, NZL suppressed pro-inflammatory cytokines such as TNF-α, IL-1β and IL-6 due to its immune regulatory function (Figure 7C). In addition, NZL significantly inhibited bacterial translocation (Figure 7B) and alleviated organ injury (Figure 8). These results suggest that NZL is expected to be a promising antimicrobial agent to treat infections caused by *S. hyicus* through more clinical trials.

In conclusion, novel AMPs were designed based on the NZ2114 template and successfully expressed in *P. pastoris*. NZL possessed the most potent antibacterial activity and reduced toxicity, and maintained excellent clinical stability. NZL may kill *S. hyicus* through action on the cell wall. Moreover, NZL also showed high efficacy in a mouse peritonitis model infected by *S. hyicus*, and this effect was superior to NZ2114 and CRO. Therefore, these results support the fact that NZL has the potential to treat *S. hyicus* infections as a therapeutic alternative to currently available antibiotics.

## 4. Materials and Methods

### 4.1. Bacterial Strains, Cell Line, and Model Animals

The bacterial strains *S. aureus* ATCC 43300 and *S. aureus* ATCC 25923 were purchased from American Type Culture Collection (ATCC). *S. hyicus* NCTC 10350 was purchased from National Collection of Type Culture (NCTC). The clinical strain of *S. hyicus* ACCC 61734 (Agricultural Culture Collection of China) was obtained from Animal husbandry and veterinary research institute (Tianjin, China). Hacat cells were purchased from Peking Union Medical College (Beijing, China). The six-week-old female ICR mice (SPF) were purchased from the Vital River Laboratories (VRL, Beijing, China). All other chemical reagents used were of analytical grade.

### 4.2. Peptide Design, Expression and Purification

In this study, a series of derivatives were designed based on the parent peptide NZ2114 via AA substitution in order to obtain more potent molecules. The main structure of NZ2114 was not changed to maintain the antimicrobial activity. Besides, we analyzed the amino acid sequences and physicochemical properties of the parent peptide NZ2114 and its derivatives through bioinformatics programs.

The recombinant vectors were constructed, linearized and transformed into *P. pastoris* X-33 for protein expression. Then, peptides were purified by the AKTA express system and confirmed by tricine-sodium dodecyl sulfate polyacrylamide gel electrophoresis (Tricine-SDS-PAGE), inhibition zone assay and matrix-assisted laser desorption/ionization–time-of-flight mass spectrometry (MALDITOF MS) [16,24]. The concentration of peptide was determined by a Bradford assay.

### 4.3. Structure Analysis

The secondary structure of peptides in different environments were analyzed by CD on a MOS-450 spectropolarimeter (Bio-Logic, Grenoble, France). The peptides were dissolved in ddH_2_O, 40 mM SDS and 50% TFE, mimicking aqueous, hydrophobic environment and microbial membrane, respectively [51]. The CD spectra was recorded over the wavelength range of 180–260 nm at 25 °C for three times [26]. In addition, the three-dimensional structure of peptides was analyzed by PyMol 2.3.

### 4.4. Antimicrobial Activity Assays

#### 4.4.1. MIC

The microbroth dilution method was used to evaluate the MIC values of new derivative peptides [52]. Briefly, a series of 10 µL 2-fold peptides (1–256 μg/mL) and 90 µL bacteria suspension (1 × 10^5^ CFU/mL in MHB) were incubated at 37 °C for 16–20 h. The MIC value was defined as the lowest concentration where no visible bacteria growth occurred after overnight incubation. CRO was used as the positive control. All assays were conducted in triplicate.

#### 4.4.2. Time-Killing Curves

Time-kill curves were performed to assess the bactericidal rates of peptides against *S. hyicus* NCTC 10350 [25]. Simply, the mid-log phase of *S. hyicus* NCTC 10350 cells were diluted to 1 × 10^5^ CFU/mL with MHB and incubated with NZL or NZ2114 with the final concentration of 1×, 2×, or 4× MIC. The samples were taken at regular intervals for colony counting. Cells treated with PBS and CRO were used as the negative control and positive control.

#### 4.4.3. Intracellular Antibacterial Activity

Hacat cells (2.5 × 10^5^ cells/mL) were dispensed into a 12-well plate for 24 h. Besides, the mid-log phase *S. hyicus* NCTC 10350 (2.5 × 10^7^ CFU/mL) were coincubated with Hacat cells for 0.5 h [53]. Then, lysostaphin was supplemented to kill extracellular bacteria. Hereafter, cells were treated with peptides or CRO for 24 h and lysed with lysis buffer. The intracellular bacteria were processed for colony counting.

### 4.5. Hemolysis, Cytotoxicity, and Stability of Peptides

#### 4.5.1. Hemolysis

The hemolytic activity of peptides against fresh mouse erythrocytes was evaluated as described previously [54]. In brief, 8% (*v*/*v*) erythrocyte solution in 0.9% NaCl was mixed with peptide solution (1–256 μg/mL) in an equal volume; then the mixtures were incubated at 37 °C for 1 h, centrifuged at 5000 rpm for 5 min and measured at 540 nm on a microplate reader. The absorbance of 0.9% NaCl (A_0_) and 0.1% Triton X-100 (A_100_) was used as controls. The hemolysis percentages of peptide were calculated by the following equation: Hemolysis (%) = [(A − A_0_)/(A_100_ − A_0_)] × 100.

#### 4.5.2. Cytotoxicity

The cytotoxicity of peptides toward Hacat cells was determined by the 3-(4,5-dimethylthiazol-2-yl)-2,5-diphenyltetrazolium bromide (MTT) assay according to a previous study [9].

#### 4.5.3. Stability

The stability of NZL or NZ2114 against *S. hyicus* NCTC 10350 was conducted by inhibition zone assays [26]. To evaluate the thermal stability, 64 μg/mL NZL or NZ2114 was incubated at different temperatures (20 °C, 40 °C, 60 °C, 80 °C, and 100 °C) for 1 h. Besides, aliquots of NZL or NZ2114 were dissolved in different pH buffers in a range from 2.0 to 10.0 at 37 °C for 3 h to analyze the pH stability. NZL or NZ2114 was mixed with pepsin or trypsin solutions at a ratio of 10:1 (*w*/*w*) to determine the protease stability. The untreated peptides and buffers alone were used as positive controls and negative controls, respectively. All assays were conducted in triplicate.

### 4.6. Membrane Permeabilization Assay

#### 4.6.1. Effects of NZL on *S. hyicus* Membrane

The membrane permeabilization assay was used to study the interaction of peptides with the *S. hyicus* membrane as depicted previously [55]. The mid-log phase of *S. hyicus* NCTC 10350 cells (1 × 10^8^ CFU/mL) were incubated with 4× MIC peptides at 37 °C for 0.5 or 2 h, respectively. Then, the *S. hyicus* cells were washed and stained by the propidium iodide (PI) at 0.5 mg/mL. The fluorescence of cells was analyzed using a FACS Calibur Flow Cytometer (BD, Franklin Lakes, NJ, USA). Cells untreated were served as the blank control.

#### 4.6.2. Effects of FITC-Labeled NZL on *S. hyicus* Membrane

The mid-log phase of *S. hyicus* NCTC10350 cells (1 × 10^8^ CFU/mL) were incubated with 4× MIC FITC-labeled peptides at 37 °C for 0.5 or 2 h, respectively. Then, the *S. hyicus* cells were washed and incubated with equal volume trypan blue (0.4%) for 10 min to quench the fluorescence of membrane-bound FITC-labeled peptides [42]. FITC-positive *S. hyicus* cells were monitored by a FACS Calibur Flow Cytometer (BD, Franklin Lakes, NJ, USA). Cells untreated with trypan blue were used as controls.

### 4.7. Morphological Observations

Electron microscopy was used to characterize morphological changes of cells after exposure to the peptides. The exponential phase *S. hyicus* NCTC 10350 cells (1 × 10^8^ CFU/mL) were incubated with 4× MIC peptides at 37 °C for 2 h. The samples were processed according to a previous study [56] and visualized on a QUANTA200 SEM (FEI, Philips, Netherlands) or a JEM1400 (JEDL, Tokyo, Japan). Cells untreated were used as the blank control.

### 4.8. Super-Resolution Microscopy Image

The localization of peptides in bacteria was observed by super-resolution microscopy [57]. The mid-log phase *S. hyicus* NCTC 10350 cells were diluted to 1 × 10^8^ CFU/mL and incubated with 4× MIC FITC-labeled peptides at 37 °C for 1 h. The cells were stained by DAPI for 2 min and washed twice. Subsequently, the cells were stained by PI for 2 min, washed twice and resuspended with PBS. A 10 μL of sample was transferred to the microscope slides and added into 2 μL antifade mounting medium; then, the mixture was covered with glass microscope slides and sealed with nail enamel and observed on a nikon N-SIM S (Tokyo, Japan).

### 4.9. Efficacy of NZL in Mice

The mice (6 mice/group) were intraperitoneally infected with the exponential phase *S. hyicus* NCTC 10350 cells (1 × 10^9^ CFU/mL, 1 mL) to establish a mouse peritonitis model [38]. Therapeutic groups were intraperitoneally treated with peptides (5 mg/kg and 10 mg/kg of body weight, 200 μL) or CRO (30 mg/kg and 60 mg/kg of body weight, 200 μL) at 2 h and 8 h post-infection. Survival of mice was recorded daily for 7 days.

Likewise, mice (15 mice/group) were injected with the exponential phase *S. hyicus* NCTC 10350 cells (1 × 10^9^ CFU/mL, 1 mL) and treated with 10 mg/kg peptides or 60 mg/kg CRO. After 2 and 24 h post-treatment, sera were collected to test the levels of cytokines using enzyme linked immunosorbent assay (ELISA) kit. To evaluate bacterial translocation, organs were harvest from mice at 24-h post-treatment for colony counting; then, organs were removed from mice at 5 d post-treatment and observed through a light microscope to assess organ injury. The uninfected mice, the treated mice with CRO or PBS were served as the blank control, positive and negative control, respectively.

### 4.10. Statistical Analysis

In all experiments, statistical analyses were assessed by one-way or two-way analysis of variance (ANOVA) followed by Dunnett’s multiple comparisons test using GraphPad Prism 7.0 (GraphPad Software, La Jolla, CA, USA). All data are presented as means ± standard deviation (SD). A *p* < 0.05 was defined as statistically significant.

## Figures and Tables

**Table 1:** Predicted physicochemical properties of NZ2114 and designed peptides and determination of MIC.

Peptide	Sequences ^a^	MW ^b^	Net Charge	Hydrophobicity	Instability Index	A1 ^c^	A2 ^d^
NZ2114	GFGCNGPWNEDDLRCHNHCKSIKGYKGGYCAKGGFVCKCY	4417.03	3	0.350	25.49	4	4
1	GFGCNGPW**S**EDD**I**RCHNHCKSIKGYKGGYCAKGGFVCKCY	4390.00	3	0.366	22.41	–	–
2	GFGCNGPW**S**EDDL**K**CHNHCKSIKGYKGGYCA**R**GGFVCKCY	4390.00	3	0.364	20.29	–	–
3	GFGCNGPW**T**EDDL**K**CHNHCKSIKGYKGGYCA**SK**GFVCKCY	4406.04	3	0.371	13.44	–	–
4	GFGCNGPW**T**EDD**IK**CHNHCKSIKGYKGGYCAKGGFVCKCY	4376.01	3	0.374	16.53	–	–
5	GFGCNGPW**T**EDD**I**RCHNHCKSIKGYKGGYCA**SK**GFVCKCY	4434.05	3	0.373	15.57	–	–
6 (NZL)	GFGCNGPW**S**EDD**IQ**CHNHCKSIKGYKGGYCAKGGFVCKCY	4361.94	2	0.386	20.52	2	1
7	GFGCNGPW**S**EDDL**Q**CHNHCKSIKGYKGGYCA**R**GGFVCKCY	4389.96	2	0.383	28.67	2	4
8	GFGCNGPW**S**EDD**I**RCHNHCKSIKGYKGGYCA**SA**GFVCKCY	4362.93	2	0.398	21.45	64	128
9	GFGCNGPW**Q**EDDL**K**CHNHCKSIKGYKGGYCA**SA**GFVCKCY	4375.97	2	0.391	19.32	4	8
10	GFGCNGPW**T**EDD**IQ**CHNHCKSIKGYKGGYCA**R**GGFVCKCY	4403.98	2	0.393	16.77	>128	>128
11	GFGCNGPW**T**EDDL**K**CHNHCKSIKGYKGGYCA**SA**GFVCKCY	4348.95	2	0.403	15.57	–	–

^a^ Changed amino acids are shown in bold and underlined. ^b^ MW, molecular weight (Da). ^c^ MIC (µg/mL) determination is described as “microbroth dilution assay” against *S. aureus* ATCC 43300; inactive peptides are indicated by “–”, tested by inhibition zone assay. ^d^ MIC (µg/mL) determination is described as “microbroth dilution assay” against *S. hyicus* ACCC 61734; inactive peptides are indicated by “–”, tested by inhibition zone assay. The key physicochemical properties of peptides, MW, net charge and instability index were analyzed by ProtParam (http://web.expasy.org/protparam/; accessed on 15 October 2020). Hydrophobicity was calculated using Heliquest (https://heliquest.ipmc.cnrs.fr/; accessed on 15 October 2020).

**Table 2:** The MIC values of peptides and CRO against bacteria.

Strains	MICs					
	CRO		NZL		NZ2114	
	μg/mL	μM	μg/mL	μM	μg/mL	μM
*S. aureus* ATCC 43300	8	12.09	2	0.46	4	0.91
*S. aureus* ATCC 25923	4	6.04	4	0.92	8	1.81
*S. hyicus* NCTC 10350	8	12.09	4	0.92	4	0.91
*S. hyicus* ACCC 61734	4	6.04	1	0.23	4	0.91

## Data Availability

All data generated or analysed during this study are included in this published article.
